# Machine Learning-Driven Prediction of Low BMD in Postmenopausal Women Using Cytokine, RANKL/OPG, and Oxidative Stress Biomarker Signatures: An Exploratory Study

**DOI:** 10.3390/biomedicines14061358

**Published:** 2026-06-16

**Authors:** Fawaz Azizieh, Sedra M AlRamadan, Mahamed G. H. Omran

**Affiliations:** 1 College of Integrative Studies, Abdullah Al Salem University, Office A38, Building 31, Khaldiya Campus, Kuwait City 72303, Kuwait; 2 Data Science and Artificial Intelligence, College of Computer and Systems Engineering, Abdullah Al Salem University, Kuwait City 72303, Kuwait; sedrahalramadan@gmail.com; 3 College of Computer and Systems Engineering, Abdullah Al Salem University, Office 31, Building 14, Khaldiya Campus, Kuwait City 72303, Kuwait; mahamed.omran@aasu.edu.kw

**Keywords:** osteoporosis, cytokines, OPG, RANKL, oxidative stress, machine learning, postmenopausal women, biomarkers

## Abstract

**Aim:** To evaluate cytokine, RANKL/OPG, and oxidative stress biomarker profiles in postmenopausal women with differing bone mineral density (BMD) and to apply machine learning (ML) models for early identification of low BMD. **Materials & Methods:** Seventy-one postmenopausal women were classified as normal BMD (N), osteopenia (OSN), or osteoporosis (OSR) using ISCD/WHO criteria. Ten cytokines, RANKL, OPG, and five oxidative stress markers were quantified. Group differences were assessed using nonparametric statistics and rank-biserial correlation. ML classifiers were developed within the PyCaret automated machine learning framework and evaluated using repeated stratified cross-validation to distinguish N vs. low BMD (L = OSN + OSR) and OSN vs. OSR using cytokine-only, bone/oxidative, and integrated biomarker panels, while SHapley Additive exPlanations (SHAP) were employed to interpret feature contributions. **Results:** Low BMD was associated with elevated pro-resorptive cytokines (TNF-α, IL-6, IL-12) and reduced anti-resorptive cytokines (IL-4, IL-10, IL-23). OPG and antioxidant enzymes (catalase, SOD2, PRX2) were significantly lower in L. Effect-size findings confirmed strong associations of IL-4, IL-10, IL-23, and OPG with normal BMD, and IL-6, IL-12p70, and TNF-α with low BMD. Logistic regression integrating cytokines with OPG and SOD2 achieved the best N vs. L performance (mean F1 ≈ 0.90, SD = 0.09). Biomarkers showed limited ability to discriminate OSN from OSR (maximum F1 ≈ 0.70, SD = 0.18). **Conclusions:** Integrating cytokine, bone-regulatory, and oxidative stress markers enhances ML-based prediction of low BMD and supports improved early osteoporosis risk stratification.

## 1. Introduction

Osteoporosis is a major global health concern, particularly among postmenopausal women, and is characterized by reduced bone mass and microarchitectural deterioration that increase the risk of fractures, disability, and mortality. Globally, an estimated 200 million women are affected, and the burden continues to rise with population aging and increased life expectancy [1,2,3]. Early risk identification is essential for preventing fractures; however, dual-energy X-ray absorptiometry (DEXA), the current diagnostic gold standard for assessing bone mineral density (BMD), has limited availability, incurs substantial costs, and provides little insight into the biological mechanisms driving disease or the risk of progression. These limitations underscore the need for predictive tools that are accessible, biologically informative, and clinically actionable.

Bone remodeling is regulated by a complex interplay of immune, hormonal, and oxidative pathways. Pro-resorptive cytokines—including TNF-α, IL-6, IL-12, and IL-17—promote osteoclast differentiation and bone resorption, whereas anti-resorptive cytokines such as IL-4, IL-10, and IL-23 counterbalance inflammation and limit osteoclastogenesis [4,5]. Dysregulation of these cytokines has been implicated in postmenopausal bone loss, and our previous work has demonstrated a pronounced pro-resorptive cytokine bias in women with low BMD [6,7]. Prior multivariate analyses further suggest that cytokine signatures can differentiate normal from low BMD phenotypes [7], yet traditional statistical approaches are often unable to model the nonlinear interactions and multidimensional patterns that arise within the immune–bone axis.

Beyond cytokines, the RANKL (Receptor Activator of Nuclear Factor kappa-B Ligand)/OPG (Osteoprotegerin) axis plays a pivotal role in bone metabolism. RANKL promotes osteoclast differentiation and activation, while OPG acts as a decoy receptor, inhibiting bone resorption. An imbalance favoring RANKL or an elevated RANKL/OPG ratio has been associated with increased bone turnover and osteoporosis risk [8]. Similarly, oxidative stress (OS) has emerged as a critical contributor to bone fragility. Reactive oxygen species (ROS) impair osteoblast function, enhance osteoclast activity, and disrupt extracellular matrix integrity. Antioxidant enzymes such as catalase, superoxide dismutases (SOD1, SOD2), and peroxiredoxins (PRX2) serve as defense mechanisms, and their reduced activity has been linked to low BMD in postmenopausal women [9]. These findings suggest that RANKL, OPG, and OS markers, alongside cytokines, may provide a more comprehensive biomarker panel for osteoporosis risk stratification.

Artificial intelligence (AI) offers powerful tools for analyzing such multidimensional biological data. Machine learning (ML) techniques can detect latent interactions, model nonlinear relationships, and generate accurate predictive algorithms using high-dimensional biomarker datasets. Previous AI applications in immunology and clinical prediction—including cancer immunotherapy response, pregnancy complications, and neurological disease prognosis—have demonstrated the value of leveraging cytokine-based signatures for disease prediction [10,11].

Recent advances in osteoporosis research further highlight the emerging role of predictive ML models. Machine learning approaches have been applied to diverse data modalities, including imaging, clinical features, and body composition metrics, to improve early detection of low BMD. For example, ML models using radiographic and demographic data have shown reasonable predictive performance for osteoporosis classification, achieving accuracies approaching 79% with interpretable decision tree frameworks [12]. Similarly, explainable ML models based on bioelectrical impedance-derived body composition parameters have demonstrated high accuracy (up to ~92%) in detecting osteopenia, underscoring the importance of integrating soft tissue composition and metabolic indicators into predictive frameworks [13]. More recently, ML models that integrate BMD measurements with clinical data and optimized feature selection strategies have achieved even higher diagnostic performance. For example, supervised learning algorithms such as support vector machines, k-nearest neighbors, and neural networks have demonstrated classification accuracies exceeding 90%, with optimized feature selection approaches achieving up to 94.3% accuracy [14]. These findings highlight the importance of combining BMD with additional patient-specific variables to overcome the limitations of traditional diagnostic approaches.

Beyond imaging and anthropometric data, recent large-scale studies have emphasized the critical contribution of inflammatory pathways, showing that ML models incorporating cytokines such as IL-6, TNF-α, and IL-1β can achieve excellent predictive performance (AUC > 0.96) across multiple skeletal sites [15].

Despite these advances, most existing models rely on single-domain inputs, such as imaging features, clinical variables, or body composition measures, limiting their biological interpretability and translational potential. Importantly, key pathogenic pathways, including inflammatory cytokine networks, oxidative stress, and bone regulatory signaling systems (e.g., RANKL/OPG), have rarely been integrated into unified predictive frameworks. This lack of multidimensional integration restricts the ability to capture the complex pathophysiology of osteoporosis, which involves interactions between immune, metabolic, and skeletal systems [6,15,16].

Accordingly, ML has been used only sparingly in osteoporosis research in a biologically integrative manner [12,13,14,15], and, to our knowledge, no study has integrated cytokines, RANKL/OPG, and oxidative stress markers into unified ML models to predict BMD status in postmenopausal women. Applying similar approaches to osteoporosis could enable early detection, personalized monitoring, and targeted interventions—shifting the paradigm from reactive to predictive medicine [11].

To address this gap, the present study aims to develop and evaluate ML-based predictive models for identifying low BMD and differentiating osteopenia from osteoporosis in postmenopausal women using an integrated biomarker panel. By systematically comparing multiple ML algorithms and feature subsets, we sought to determine whether combining cytokine, bone-regulatory, and oxidative stress markers improves predictive performance beyond individual biomarker categories. Ultimately, our goal is to provide a biologically informed, data-driven framework that supports early detection, personalized risk stratification, and future translational applications in osteoporosis management.

## 2. Materials and Methods

### 2.1. Study Design and Population

**Figure 1:**
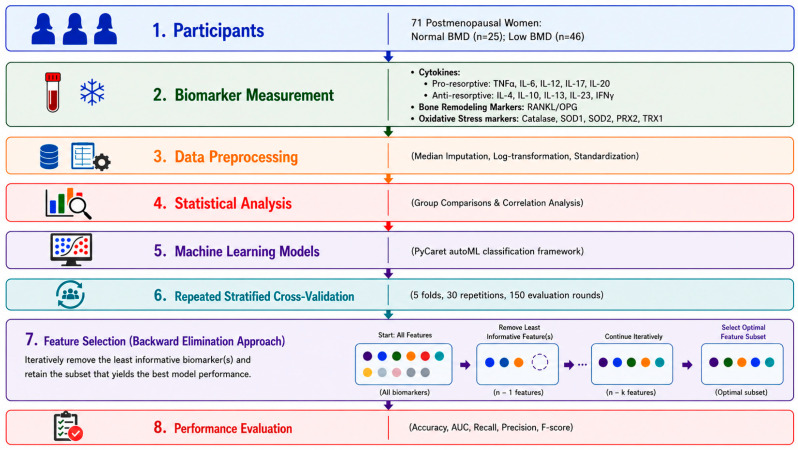
Schematic representation of the study workflow.

An overview of the study workflow is provided in Figure 1. This study analyzed data from 71 postmenopausal women, previously described in our earlier publications on cytokine profiles and bone health [6,16]. Inclusion criteria were: absence of menstrual periods for at least 12 months prior to enrollment, and no history of systemic corticosteroid use, malignancy, hyperparathyroidism, severe renal or hepatic impairment, or active infection. Demographic data (age, BMI, years since menopause) were recorded at the time of examination. Participants were recruited from the Physical Medicine Unit at Mubarak Al Kabeer Hospital, Kuwait.

### 2.2. Grouping and Bone Mineral Density Assessment

Bone mineral density (BMD) was measured at the lumbar spine (L1–L4) and left hip using dual-energy X-ray absorptiometry (DEXA) (GE Lunar, Madison, WI). Diagnosis was based on the guidelines set by the WHO and Adult Official Positions of the International Society for Clinical Densitometry (ISCD). Participants were grouped as women with normal BMD (N, n = 25) or with low BMD (L, n = 46). Participants were further grouped by the T-scores of BMD into three groups: the normal group (N, T-scores ≥ −1, n = 25), the osteopenia group (OSN, −2.5 < T-scores < −1, n = 31) and the osteoporosis group (OSR, T-scores ≤ −2.5, n = 15).

### 2.3. Biomarker Measurement

Peripheral blood samples were collected and processed as previously described [6,16]. Analyte measurements were as follows:

- Cytokines: Levels of pro-resorptive (TNF-α, IL-6, IL-12, IL-17, IL-20) and anti-resorptive (IFN-γ, IL-4, IL-10, IL-13, IL-23) cytokines were measured in mitogen-stimulated PBMC supernatants using kits from Merck Millipore, Darmstadt, Germany (Cat.# HCYTOMAG-60K) using multiplex ELISA (Luminex platform; Austin, TX, USA).
- Bone Remodeling Markers: Serum levels of RANKL and OPG quantified using MILLIPLEX MAP assays (Merck Millipore; Cat.# HRNKLMAG-51K and HBNMAG-51K).
- Oxidative Stress Markers: Serum levels of Catalase, PRX2, SOD1, SOD2, and TRX1 measured using Human Oxidative Stress Magnetic Bead Panel (Merck Millipore, Cat.# H0XSTMAG-18K).

Assay sensitivities and quality control procedures followed manufacturer guidelines. All measurements were performed in duplicate.

### 2.4. Data Preprocessing

Undetectable cytokine values were replaced with assay sensitivity thresholds; missing values were replaced with the median substitution, which is a conservative technique, not biased towards specific characteristics of the data. All variables were subsequently log-transformed as part of the standard preprocessing steps to stabilize variance and reduce skewness. Finally, the data were centered (by subtracting the mean) and scaled (by dividing by the standard deviation) so that each variable has a mean of 0 and a standard deviation of 1. Ensuring that all variables are on the same scale is particularly crucial to facilitate easy and meaningful comparison between variables. Outliers were assessed visually and retained to preserve biological variability.

### 2.5. Statistical Analysis

Normality of data distribution was assessed using the Shapiro–Wilk test. Most variables exhibited non-normal distributions; therefore, the nonparametric Mann–Whitney U test was applied for group comparisons. For variables that met normality assumptions, independent samples *t*-tests were used. Significance threshold is set to *p* < 0.05. Analyses were performed using SPSS v23 and SciPy.stats v1.15 in Python.

### 2.6. Effect Size

After establishing statistical significance, reporting effect size is essential to quantify the practical importance of the observed differences. Statistical significance indicates whether an effect is unlikely to have occurred by chance, but it does not convey the magnitude or relevance of that effect. Effect size measures provide information about the strength and direction of the relationship or group difference, allowing results to be interpreted in a meaningful and comparable way across studies. Consequently, effect size complements hypothesis testing by supporting more robust, transparent, and scientifically informative conclusions [17].

To quantify the effect size of the difference between the normal (N) and low (L) BMD groups, and to further characterize the magnitude of differences in variable values between these two groups, the rank-biserial correlation (r_rb_) was employed [18]. This statistic is a nonparametric, rank-based measure of effect size that assesses the association between a binary variable (group membership: N = 1, L = 0) and a continuous outcome. The rank-biserial correlation provides an interpretable measure of the strength and direction of the group difference and is commonly used to express the magnitude of the effect identified by the Mann–Whitney U test. The r_rb_ ranges from −1 to +1:

- r_rb_ = 0: no association (distributions largely overlap).
- r_rb_ > 0: values in Normal BMD group tend to be higher than in Low BMD.
- r_rb_ < 0: values in Low BMD group tend to be higher than in Normal BMD.

For interpreting |r_rb_|: ≈0.10 → Small effect, ≈0.30 → Medium effect, and ≥0.50 → Large effect.

### 2.7. Machine Learning Techniques

In this study, multiple machine learning-based binary classification models from the PyCaret AutoML package version 3.3.2 [19,20] were evaluated to distinguish between the normal BMD and low BMD groups. PyCaret evaluates and ranks a wide range of classification algorithms, including Logistic Regression, SVM with a linear kernel, Extra Trees Classifier, Naive Bayes, Ridge Classifier, Gradient Boosting Classifier, Quadratic Discriminant Analysis, Linear Discriminant Analysis, Decision Tree Classifier, K Neighbors Classifier, Light Gradient Boosting Machine, Random Forest Classifier, and Ada Boost Classifier. The performance of the different models has been measured using relevant metrics like accuracy, Area Under the Curve (AUC), recall, precision, and F1 score. The F1 Score, which is the harmonic mean between recall and precision, is the most appropriate measure when the dataset is imbalanced, as in our case [17]. It provides a balanced assessment of a model’s effectiveness, penalizing models that perform poorly in either precision (the proportion of predicted positives that are truly positive) or recall (the proportion of actual positives that are correctly identified). The accuracy metric is reported to indicate the ratio of the number of correct predictions over total predictions. The Area Under the Curve (AUC) is used as a statistic measuring the quality of the scoring function defining the classifier. The AUC metric ranges between 0 and 1, with scores close to 1 indicating near-perfect classification ability.

To obtain a reliable estimate of model performance under the constraints of the limited sample size available in this study, repeated stratified k-fold cross-validation was employed during model training and evaluation. Specifically, a 5-fold stratified cross-validation procedure was repeated 30 times, each repetition using a different random partitioning of the data, yielding 150 evaluation rounds per model. Stratification was applied at every fold to preserve the class distribution of the original dataset across all training and test partitions. Performance metrics were aggregated across all 150 rounds and reported as mean (SD), capturing both the central tendency and the variability of model performance across the repeated evaluations. This strategy was selected over a single-run cross-validation approach as it substantially reduces the sensitivity of performance estimates to any particular random split of the data.

To enhance the interpretability of the best-performing model, SHapley Additive exPlanations (SHAP) analysis was conducted using the KernelExplainer, a model-agnostic explainer applicable to probabilistic classifiers. SHAP values provide a consistent and interpretable measure of feature importance by attributing each prediction to the contribution of individual features, allowing identification of the most influential predictors and their directional impact on model output [21]. SHAP was applied to the standardized input data used during model training to ensure consistency in feature representation, and values were computed for all training samples. Results were visualized as a mean absolute feature importance bar plot and a summary dot plot, together conveying the magnitude and direction of each feature’s contribution to the model’s predictions.

### 2.8. Feature Selection

Feature selection was performed using a wrapper-based backward elimination strategy to evaluate the predictive contribution of different biomarker domains while identifying reduced feature subsets that improved classification performance.

The primary objective of feature selection was to determine whether reducing the number of input variables could improve model generalizability and interpretability without compromising predictive accuracy. Preliminary univariate analyses identified features showing significant differences between BMD groups, offering an initial view of potential biological drivers. To complement these findings, machine learning models were used to assess the joint contribution of all features simultaneously. This distinction is important: whereas univariate tests evaluate each marker in isolation, multivariate models capture synergistic effects, nonlinear interactions, and unique variance that may not be apparent individually [22]. Consequently, biomarkers that were not individually significant were retained when they improved multivariate classification performance, whereas statistically significant biomarkers could be excluded if their information was redundant in the presence of other correlated variables. This complementary strategy underscores the importance of integrating statistical inference with machine learning to reveal subtle but biologically meaningful patterns. Three feature sets were analyzed sequentially:

- Cytokine panel only, comprising pro-resorptive (TNF-α, IL-6, IL-12, IL-17, IL-20) and anti-resorptive (IFN-γ, IL-4, IL-10, IL-13, IL-23) cytokines.
- Bone- and oxidative-stress biomarkers, comprising RANKL, OPG, and the five oxidative stress markers.
- Integrated multi-biomarker panels combining cytokines with bone-related and oxidative-stress markers.

For each feature set, an iterative backward elimination procedure was applied. Model performance was first evaluated using the complete feature set. At each elimination step containing k features, k candidate subsets were generated by removing one feature at a time. Each candidate subset was then evaluated by re-executing the complete machine-learning pipeline and estimating performance using repeated stratified 5-fold cross-validation (30 repetitions). The feature whose removal resulted in the greatest performance improvement, or the smallest performance degradation, was permanently excluded. This process was repeated iteratively until no further feature removal improved model performance or until additional feature elimination resulted in unacceptable performance loss. Importantly, the feature-selection procedure employed a greedy search strategy rather than an exhaustive combinatorial search. For example, an exhaustive search of the integrated panel containing 17 features would require evaluation of 2^17^ (131,072) possible feature subsets. In contrast, the backward elimination procedure evaluates only the immediate candidate subsets available at each iteration, substantially reducing the search space and limiting the risk of overfitting associated with exhaustive subset exploration in small datasets. Feature selection was conducted separately for each classification task:

- Normal vs. Low BMD.
- Osteopenia vs. Osteoporosis within the Low BMD subset.

For each biomarker panel and classification task, multiple candidate feature subsets were evaluated during the elimination process. To maintain clarity and conciseness, only the final feature subsets selected by the backward elimination procedure, corresponding to the highest cross-validated performance, are presented in Section 3.

This strategy enabled assessment of both the independent predictive value of each biomarker domain and the incremental benefit obtained by integrating inflammatory, bone-metabolism, and oxidative-stress markers within a unified predictive framework. Because of the limited sample size (n = 71), feature selection and performance estimation were both conducted using repeated cross-validation on the available dataset. Specifically, cross-validated performance estimates were used to guide the feature-selection process, meaning that feature selection was not performed within a nested cross-validation framework. Consequently, the reported performance metrics may be subject to a degree of optimistic bias and should be interpreted as estimates of predictive performance rather than fully independent measures of external generalizability. Therefore, the identified biomarker signatures should be considered exploratory and hypothesis-generating until validated in larger independent cohorts.

## 3. Results

### 3.1. Participant Characteristics

Demographic and clinical characteristics of the study population are summarized in Table 1. Women with low BMD (L) were slightly older than those with normal BMD (N) (*p* = 0.03), and age also differed between the N and OSR subgroups (*p* = 0.001), whereas BMI and years since menopause did not differ significantly between groups. As expected, comparisons across the three diagnostic subcategories (N, OSN, OSR) showed stepwise reductions in hip and spine BMD and T-scores. Given the potential for confounding, prior regression analysis [6] was conducted to assess the influence of age and years since menopause on BMD outcomes. In the present study, however, age was deliberately excluded as a feature from the machine learning (ML) models. This design choice was made to specifically evaluate the independent predictive performance of cytokines and biochemical markers, without conflation from well-established clinical determinants such as age. Accordingly, the ML predictions reported here reflect the standalone discriminatory capacity of the biomarker panel, rather than age-adjusted models.

### 3.2. Comparative Analysis of Cytokines, Bone Remodeling, and Oxidative Stress Markers Across Study Groups

#### 3.2.1. Cytokine Profiles

**Figure 2:**
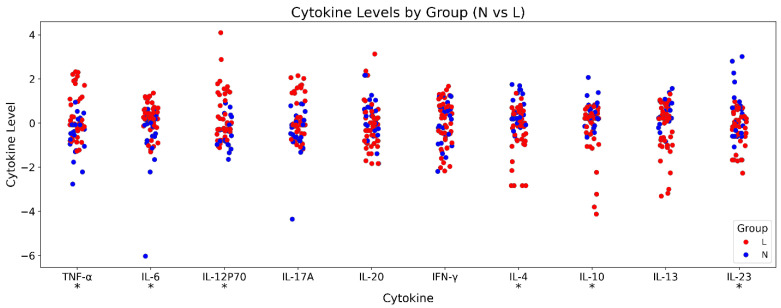
Cytokine expression profiles across groups N (blue) and L (red). Asterisks denote statistically significant differences between groups (*p* < 0.05).

Figure 2 summarizes the distribution of cytokine responses in mitogen-stimulated PBMC cultures across the N and L groups. Compared with women with normal BMD, those in the low BMD group exhibited significantly higher levels of the pro-resorptive cytokines TNF-α (*p* < 0.021), IL-6 (*p* < 0.01), and IL-12 (*p* < 0.013). In contrast, the anti-resorptive cytokines IL-4 (*p* < 0.004), IL-10 (*p* < 0.027), and IL-23 (*p* < 0.029) were significantly lower in the low BMD group. No cytokine showed a significant difference between the osteopenia (OSN) and osteoporosis (OSR) subgroups.

#### 3.2.2. Bone Remodeling Markers

**Figure 3:**
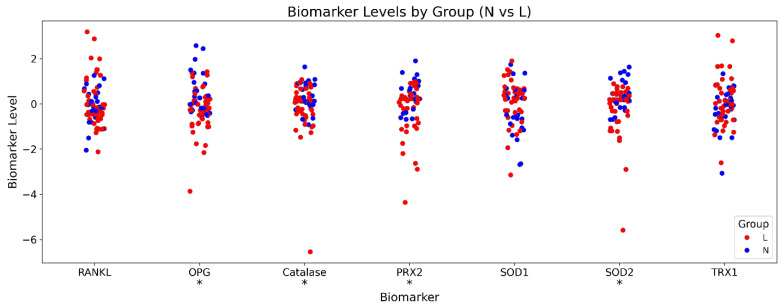
Profiles of bone remodeling markers (OPG & RANKL), and oxidative stress markers (PRX2, SOD1, SOD2, TRX1, and Catalase) across groups N (blue) and L (red). Asterisks denote statistically significant differences between groups (*p* < 0.05).

RANKL concentrations did not differ significantly among groups. OPG levels, however, were markedly higher in women with normal BMD compared with those with low BMD (*p* < 0.006) (Figure 3). No significant differences in RANKL or OPG were observed between OSN and OSR.

#### 3.2.3. Oxidative Stress Markers

Women with low BMD exhibited significantly lower serum levels of the antioxidant enzymes catalase (*p* = 0.024), SOD2 (*p* = 0.009), and PRX2 (*p* = 0.011) compared with normal BMD subjects (Figure 3). SOD1 and TRX1 displayed no significant between-group differences. As with cytokines and bone markers, oxidative stress profiles did not differ significantly between the OSN and OSR subgroup.

### 3.3. Effect Size Analysis

**Figure 4:**
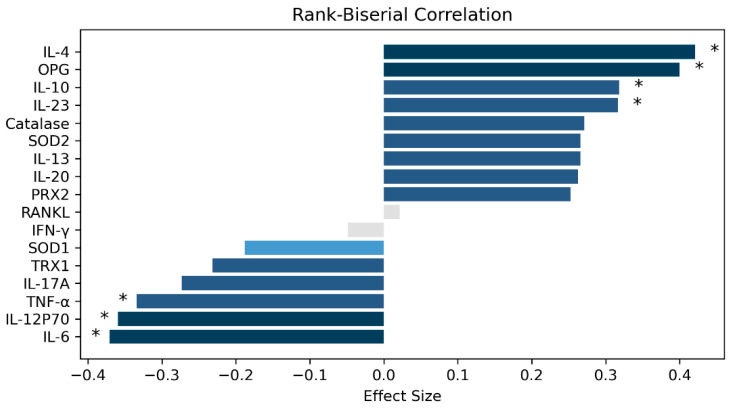
Rank-Biserial correlation coefficients for the features associated with BMD status. Positive values indicate higher levels in individuals with normal BMD (Class 1), while negative values reflect higher levels in those with low BMD (Class 0). Asterisks denote statistically significant associations (*p* < 0.05).

Rank-biserial correlation (r_rb_) values were calculated to assess the magnitude and direction of group differences (Figure 4). IL-4 (r_rb_ = 0.42, *p* = 0.0036), OPG (r_rb_ = 0.40, *p* = 0.0057), IL-10 (r_rb_ = 0.32, *p* = 0.0270), and IL-23 (r_rb_ = 0.32, *p* = 0.0286) showed the strongest positive associations with BMD. Conversely, IL-6 (r_rb_ = –0.37, *p* = 0.0103), IL-12p70 (r_rb_ = –0.36, *p* = 0.0130), and TNF-α (r_rb_ = –0.33, *p* = 0.0208) were most strongly negatively associated with BMD. These effect size findings corroborate the univariate statistical comparisons.

### 3.4. Machine Learning Classification Performance

#### 3.4.1. Prediction of Normal vs. Low BMD

##### Cytokines Only

Using all 10 cytokines as input features, Logistic Regression achieved the highest classification performance (mean F1-score = 0.81, SD = 0.12; AUC = 0.91, SD = 0.09) (Table 2, Ia). Reducing the cytokine panel to five markers (TNF-α, IL-10, IL-6, IL-4, IL-23) improved performance further, yielding an optimized mean F1-score of 0.83, SD = 0.12 (Table 2, Ib).

##### Bone Remodeling + Oxidative Stress Markers

Models trained using RANKL, OPG, and the five oxidative stress biomarkers showed slightly lower predictive power compared with cytokines. The Gradient Boosting Classifier performed best (mean F1 = 0.74, SD = 0.12) (Table 2, IIa). Dimensionality reduction identified a four-marker subset (OPG, SOD1, SOD2, PRX2) that improved performance with Logistic Regression achieving a mean F1 = 0.77, SD = 0.12, indicating these markers capture the core discriminative signal (Table 2, IIb).

##### Integrated Feature Sets

Combining the refined cytokine subset with two key biomarkers (OPG and SOD2) resulted in the highest-performing model. Logistic Regression achieved a mean F1-score of 0.90, SD = 0.09 and an AUC of 0.93, SD = 0.08, outperforming all other models and feature sets. This integrated panel provided the most robust separation between normal and low BMD groups (Table 2, III).

##### SHAP-Based Model Interpretation

**Figure 5:**
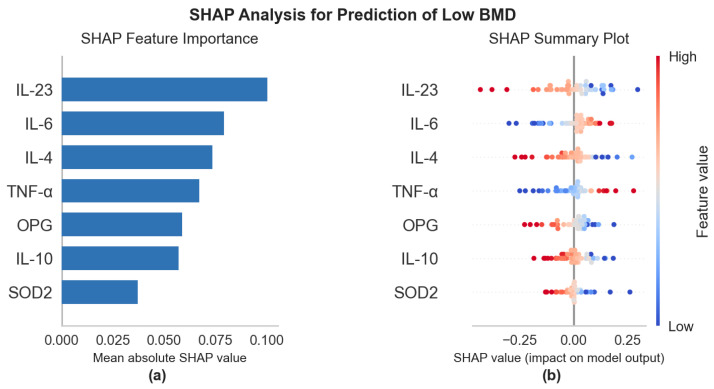
SHAP-based feature importance analysis for the Low BMD prediction task using the best-performing classification model. (**a**) Bar plot showing mean absolute SHAP value for each feature, ranked according to its overall contribution to model predictions. (**b**) Dot summary plot showing the distribution and direction of SHAP values across all training samples. Each dot represents an individual sample; dot color indicates the standardized feature value (red = high, blue = low). Positive SHAP values indicate a contribution toward the prediction of the Low BMD class, whereas negative SHAP values indicate a contribution toward the prediction of the Normal BMD class.

The SHAP analysis applied to the best-performing Logistic Regression model provides insight into both the overall importance of the features and the direction of their influence on the prediction of the Low BMD class (Figure 5). The feature importance plot (Figure 5a) indicates that IL-23 was the most influential predictor, exhibiting the highest mean absolute SHAP value, followed by IL-6, IL-4, and TNF-α. OPG and IL-10 showed moderate contributions, whereas SOD2 had the smallest overall impact on model predictions. The SHAP summary plot (Figure 5b) further reveals the direction of each feature’s effect on model predictions. For IL-23, higher feature values (red points) were predominantly associated with negative SHAP values, while lower values (blue points) were associated with positive SHAP values. This pattern indicates that lower IL-23 levels increased the likelihood of a sample being classified as Low BMD. A similar inverse relationship was observed for IL-4, OPG, IL-10, and SOD2, where lower feature values generally contributed toward the prediction of the Low BMD class. In contrast, IL-6 and TNF-α exhibited the opposite trend. Higher IL-6 and TNF-α levels were associated with positive SHAP values, indicating an increased contribution toward the prediction of the Low BMD class. This pattern is consistent with the findings of the preliminary statistical analysis, which likewise demonstrated elevated concentrations of these biomarkers in individuals with low BMD.

Overall, the SHAP analysis highlights IL-23, IL-6, and IL-4 as the most influential features in the Logistic Regression model and demonstrates that both increased pro-inflammatory signaling (e.g., IL-6 and TNF-α) and reduced levels of certain regulatory biomarkers (e.g., IL-23, IL-4, OPG, IL-10, and SOD2) contributed to the prediction of Low BMD. These results provide biologically interpretable evidence supporting the model’s decision-making process.

#### 3.4.2. Prediction of Osteopenia vs. Osteoporosis

Classification performance within the low BMD subgroup (OSN vs. OSR) was generally modest across all feature sets. The best-performing model (Naive Bayes Classifier using IL-4, IFN-γ, SOD2, and TRX1) achieved a mean F1-score of 0.70, SD = 0.18. Most models displayed a tendency to misclassify osteoporosis cases as osteopenia, reflecting class imbalance and limited biomarker separability between these clinically adjacent groups (Table 3).

As a summary, across biomarker domains, women with low BMD exhibited a consistent pattern characterized by (1) elevated pro-resorptive cytokines, (2) reduced anti-resorptive cytokines and OPG, and (3) lower antioxidant enzyme levels. These molecular differences enabled high-accuracy discrimination of normal vs. low BMD through machine learning models, particularly when cytokines and select biomarkers were integrated. In contrast, osteopenia and osteoporosis subgroups showed minimal biological separability, resulting in markedly lower classification performance.

## 4. Discussion

In this study, we investigated the immunological, bone-regulatory, and oxidative stress signatures associated with BMD status in postmenopausal women and evaluated their utility in machine learning-based predictive modeling. The combined biomarker and ML-driven approach enabled a comprehensive assessment of the molecular alterations accompanying low BMD and facilitated precise discrimination between normal and low BMD groups. These findings build upon previous work demonstrating cytokine dysregulation in postmenopausal bone loss and extend the field by integrating multi-domain biomarkers into predictive models with high accuracy [4,5,6,16].

The cytokine findings reaffirm the pivotal role of immune dysregulation in osteoporosis. Women with low BMD exhibited a prominent pro-resorptive cytokine milieu characterized by elevated TNF-α, IL-6, IL-12, and IL-17. These cytokines converge mechanistically to enhance osteoclastogenesis, amplify RANKL signaling, and accelerate bone resorption—mechanisms consistently implicated in postmenopausal skeletal decline [4,5]. Conversely, IL-4, IL-10, and IL-23—cytokines known to counteract inflammatory activation, suppress osteoclast differentiation, and support immune homeostasis—were significantly reduced in the low BMD group. The direction and magnitude of effect sizes strongly support this dichotomous cytokine pattern, indicating a shift toward a chronically pro-resorptive immune environment in women with low BMD [6,7].

These observations align with contemporary literature identifying immune-mediated mechanisms as central drivers of postmenopausal osteoporosis and emphasize the relevance of systemic inflammatory balance in skeletal homeostasis. Moreover, the lack of significant cytokine differences between osteopenia and osteoporosis suggests that inflammatory dysregulation may emerge early in bone loss and plateau with progression, limiting its ability to distinguish severity levels within the low BMD spectrum.

The RANKL–OPG axis remains a cornerstone in understanding bone remodeling in osteoporosis [8,16]. Although RANKL levels did not differ significantly across groups, OPG concentrations were predictably higher in women with normal BMD. Reduced OPG in low BMD subjects may reflect impaired compensatory buffering against RANKL-mediated osteoclast activation. This finding, combined with elevated pro-resorptive cytokines, reinforces the multifactorial deregulation of osteoclastogenic pathways associated with low BMD. While the RANKL/OPG ratio is widely recognized as a composite index of bone turnover, we deliberately modeled RANKL and OPG as independent features within the machine learning (ML) framework. Including the ratio alongside its individual components would introduce multicollinearity due to its mathematical dependence on these variables, potentially inflating model variance and obscuring feature-specific contributions. Notably, OPG alone demonstrated a significant association with low BMD (*p* = 0.006), indicating that its predictive signal is already captured without requiring a derived ratio. Moreover, given the modest sample size, feature dimensionality was intentionally constrained to reduce overfitting risk. To further assess this methodological choice, we conducted a supplementary analysis in which the RANKL/OPG ratio replaced its individual components. This approach resulted in lower predictive performance (Gradient Boosting Classifier F-score = 0.70, SD = 0.14) compared with the original feature structure retaining separate inputs (F-score = 0.74, SD = 0.12), supporting the decision to preserve feature independence in the final model.

Oxidative stress markers further differentiated normal from low BMD groups, with catalase, SOD2, and PRX2 showing substantial reductions in low BMD individuals. These enzymes mitigate reactive oxygen species (ROS), which themselves promote osteoclast activity, inhibit osteoblast differentiation, and impair extracellular matrix integrity [9,16]. Their diminished levels support growing evidence that oxidative stress contributes meaningfully to skeletal fragility in postmenopausal women. As with cytokines, the similarity of oxidative stress profiles between osteopenia and osteoporosis highlights the limited phenotypic separation between these adjacent diagnostic categories.

A major strength of this study is the integration of multi-biomarker profiles with machine learning (ML) to improve the prediction of low BMD. Cytokines alone demonstrated strong predictive ability, consistent with their marked biological differences between groups. Bone remodeling and oxidative stress markers provided moderate discriminatory power, but the combination of cytokines with key biomarkers—specifically OPG and SOD2—yielded a substantial boost in classification performance. The logistic regression model trained on this integrated feature set achieved a mean F1-score of approximately 0.90, SD = 0.09 across cross-validation folds, outperforming all other models and confirming the additive value of integrating inflammatory, bone-regulatory, and oxidative domains.

The identified key predictors—IL-10, IL-23, IL-4, TNF-α, IL-6, OPG, and SOD2—represent a biologically coherent panel. Together, they reflect the interplay between inflammation, bone turnover, and oxidative stress, and emphasize the multifactorial nature of bone loss in postmenopausal women. The ability of ML models to capture nonlinear interactions among these pathways underscores their potential to augment traditional diagnostic strategies.

In contrast, classification of osteopenia vs. osteoporosis demonstrated substantially lower accuracy across all feature sets. This outcome aligns with the minimal biomarker differences observed between these groups and highlights the biological overlap and clinical challenges in discriminating borderline bone phenotypes. Biomarker levels appear to plateau across these adjacent disease states, suggesting that differentiating osteopenia from osteoporosis may require the inclusion of dynamic bone turnover markers, advanced imaging modalities, or analyses based on larger, more balanced cohorts. Class imbalance further contributed to model underperformance, as osteoporosis cases were frequently misclassified as osteopenia.

This study benefits from a comprehensive biomarker panel spanning inflammation, bone remodeling, and oxidative stress pathways, combined with rigorous statistical analysis and systematic application of multiple ML algorithms. This multi-domain signature may serve as a foundation for predictive modeling and targeted interventions, including biologics or immunomodulatory strategies to restore the balance between bone formation and resorption.

Nonetheless, several limitations must be acknowledged. First, the relatively small sample size and class imbalance, particularly within the osteoporotic subgroup, may limit generalizability and reduce sensitivity for detecting subtle effects. In addition, because feature selection and model evaluation were both performed using repeated cross-validation on the same dataset rather than within a nested cross-validation framework, the reported performance estimates may be subject to optimistic bias. Consequently, metrics such as accuracy, AUC, and F1 score may overestimate performance that would be achieved on an independent external dataset. Therefore, the reported results should be interpreted with caution and considered exploratory. While similar cohort sizes are frequently reported in early-stage or proof-of-concept machine learning studies in osteoporosis prediction [12,13,14,15], such designs primarily aim to demonstrate feasibility rather than establish definitive clinical performance. Accordingly, our findings require validation in larger, well-balanced cohorts.

Second, the cross-sectional design precludes establishing causal relationships or evaluating biomarker dynamics over time, limiting the ability to assess prediction of incident osteoporosis or fracture outcomes. Future studies should incorporate longitudinal designs to evaluate biomarker stability and temporal predictive performance.

An important methodological consideration is that cytokines were measured in mitogen-stimulated PBMC supernatants, whereas bone turnover and oxidative stress markers were assessed in serum [6,16]. The use of mixed biological compartments may limit direct comparability across biomarkers and complicate interpretation, particularly in the context of clinical translation where serum-based measurements are more commonly utilized.

Furthermore, although external validation represents the gold standard for assessing model generalizability, assembling an independent cohort was not feasible within the scope of this study. This reflects both the limited availability of patients meeting the inclusion criteria and the logistical and resource constraints associated with comprehensive biochemical profiling. Consequently, independent validation in external populations remains an essential next step.

Future studies should include larger, multicenter and longitudinal cohorts, expand biomarker domains (e.g., metabolomic, genomic, or microbiome-derived features), and integrate imaging data alongside established clinical risk factors. Such approaches may enhance model robustness and support the development of clinically deployable, machine learning-driven tools for early osteoporosis risk stratification.

## 5. Conclusions

This study demonstrates that integrating cytokine, bone-regulatory, and oxidative stress markers provides a robust biomarker signature of low bone mineral density in postmenopausal women. Machine-learning models leveraging this multi-domain panel—particularly IL-10, IL-23, IL-4, TNF-α, IL-6, OPG, and SOD2—achieved high accuracy in distinguishing normal from low BMD, underscoring the value of biologically informed feature integration. In contrast, osteopenia and osteoporosis showed limited biomarker separability, reflecting their biological overlap and the challenge of differentiating these adjacent stages. This pattern indicates that the biomarker panel is more effective for identifying low BMD as a unified condition rather than distinguishing disease severity.

These findings highlight the potential of ML-driven biomarker profiling to support early detection and personalized risk stratification in osteoporosis, especially in settings where DEXA access is limited. Validation in larger, independent, and longitudinal cohorts will be essential to confirm generalizability and to advance these integrated models toward clinical translation.

Clinical Assessment of Usefulness and Potential Impact: The clinical implications of this work are substantial, particularly for settings where access to DEXA scanning is limited or delayed. By integrating cytokine, RANKL/OPG, and oxidative-stress biomarkers, the proposed ML model provides a biologically grounded tool capable of identifying women at risk of low BMD with high accuracy. The integrated logistic regression model (mean F1 ≈ 0.90, SD = 0.09) demonstrates potential as an adjunct clinical decision-support system that can flag high-risk individuals earlier in the care pathway, prompting timely referral for imaging, lifestyle intervention, or pharmacologic evaluation. Because the required biomarkers can be measured from standard blood samples, this approach could enhance osteoporosis screening in primary-care, endocrinology, and women’s-health clinics, especially in regions with limited imaging capacity. Furthermore, by capturing immune, skeletal, and oxidative signatures that precede structural bone loss, the model may support risk stratification before fractures occur, ultimately reducing morbidity, healthcare utilization, and long-term disability among postmenopausal women. Collectively, these findings highlight a clinically actionable framework for augmenting current osteoporosis assessment protocols through ML-driven biomarker profiling. While the integrated biomarker model demonstrated strong predictive performance within this cohort, these findings should be interpreted cautiously given the relatively small and imbalanced sample. External validation in larger, independent populations is required before clinical implementation.

## Figures and Tables

**Table 1:** Demographic data and baseline clinical characteristics of postmenopausal women enrolled in the study presented as mean (SD). Significant *p*-values are depicted in bold.

	Normal BMD	Low BMD	*p*	Osteopenia	*p*	Osteoporosis	*p*	*p*
	(N) (n = 25)	(L) (n = 46)	(N vs. L)	(OSN) (n = 31)	(N vs. OSN)	(OSR) (n = 15)	(N vs. OSR)	(OSN vs. OSR)
Age (years)	56.1 (5.6)	59.6 (7.8)	**0.03**	58.7 (7.9)	0.16	61.3 (7.3)	**0.001**	0.14
Weight (Kg)	80.5 (12.2)	75.5 (12.7)	0.13	75.7 (12.1)	0.55	75.2 (12.3)	0.18	0.96
Height (m)	158.7 (5.2)	156.1 (6.1)	0.15	157.3 (5.3)	0.18	153.5 (6.9)	**0.02**	0.58
BMI (kg/m^2^)	32.0 (5.2)	31.0 (5.0)	0.47	30.6 (5.0)	0.28	31.8 (5.1)	0.9	0.4
Years since menopause (yr)	7.6 (5.5)	9.0 (7.2)	0.53	7.4 (6.4)	0.75	12.1 (7.7)	**0.06**	**0.019**
T-score hip	0.2 (0.8)	−1.4 (0.87)	**0.0001**	−1.1 (0.8)	**0.0001**	−2.0 (0.7)	**0.0001**	**0.001**
Hip BMC (g/cm^2^)	1.02 (0.12)	0.78 (0.12)	**0.0001**	0.81 (0.13)	**0.0001**	0.73 (0.1)	**0.0001**	0.074
T-score L1-L4	−0.11 (0.65)	−2 (0.65)	**0.0001**	−1.7 (0.4)	**0.0001**	−2.7 (0.5)	**0.0001**	**0.0001**
L1-L4 BMC (g/cm^2^)	1.17 (0.1)	0.90 (0.1)	**0.0001**	0.93 (0.09)	**0.0001**	0.83 (0.9)	**0.0001**	**0.003**

**Table 2:** Comparative performance of the top three machine learning models in classifying Normal vs. Low BMD groups using different features presented as mean (SD).

Model	Accuracy	AUC	Recall	Precision	F1-Score
**Ia. All 10 Cytokines**					
Logistic Regression	**0.81 (0.11)**	**0.91 (0.09)**	**0.82 (0.11)**	**0.83 (0.12)**	**0.81 (0.12)**
K-Neighbors Classifier	0.77 (0.13)	0.84 (0.12)	0.77 (0.13)	0.80 (0.13)	0.77 (0.13)
Ridge Classifier	0.77 (0.13)	0.86 (0.11)	0.77 (0.13)	0.80 (0.14)	0.77 (0.14)
**Ib. Subset of cytokines: TNF**-**α, IL-10, IL-6, IL-4, IL-23**					
Logistic Regression	**0.84 (0.11)**	**0.93 (0.08)**	**0.84 (0.11)**	**0.86 (0.12)**	**0.83 (0.12)**
SVM (Linear Kernel)	0.82 (0.11)	0.91 (0.09)	0.82 (0.11)	0.84 (0.12)	0.81 (0.12)
Linear Discriminant Analysis	0.82 (0.12)	0.89 (0.11)	0.82 (0.12)	0.83 (0.13)	0.81 (0.13)
**IIa. Bone Remodeling + Oxidative Stress Markers**					
Gradient Boosting Classifier	**0.75 (0.12)**	**0.79 (0.14)**	**0.75 (0.12)**	**0.77 (0.13)**	**0.74 (0.12)**
Logistic Regression	0.74 (0.12)	0.82 (0.14)	0.74 (0.12)	0.75 (0.14)	0.73 (0.13)
Ridge Classifier	0.72 (0.12)	0.82 (0.14)	0.72 (0.12)	0.74 (0.14)	0.71 (0.13)
**IIb. Subset of biomarkers: OPG, SOD1, SOD2, PRX2**					
Logistic Regression	**0.78 (0.12)**	0.87 (0.11)	**0.78 (0.12)**	**0.80 (0.13)**	**0.77 (0.12)**
Ada Boost Classifier	0.78 (0.11)	0.80 (0.15)	0.78 (0.11)	0.79 (0.13)	0.76 (0.12)
Ridge Classifier	0.77 (0.12)	**0.87 (0.11)**	0.77 (0.12)	0.79 (0.13)	0.76 (0.13)
**III. Integrated Feature Sets: TNF**-**α, IL-10, IL-6, IL-4, IL-23, OPG, SOD2**					
Logistic Regression	**0.90 (0.08)**	**0.93 (0.08)**	**0.90 (0.08)**	**0.91 (0.08)**	**0.90 (0.09)**
SVM (Linear Kernel)	0.84 (0.10)	0.91 (0.09)	0.84 (0.10)	0.86 (0.11)	0.83 (0.11)
Ridge Classifier	0.84 (0.12)	0.89 (0.11)	0.84 (0.12)	0.85 (0.13)	0.83 (0.13)

**Table 3:** Comparative performance of the best machine learning model in classifying Osteopenia vs. Osteoporosis groups using different features presented as mean (SD).

Model	Accuracy	AUC	Recall	Precision	F1-Score
**Ia. All 10 Cytokines**					
K-Neighbors Classifier	0.67 (0.15)	0.57 (0.21)	0.67 (0.15)	0.64 (0.19)	0.64 (0.16)
**Ib. Subset of cytokines: IFN**-**γ, IL-4-23**					
K-Neighbors Classifier	0.73 (0.13)	0.61 (0.21)	0.73 (0.13)	0.68 (0.19)	0.69 (0.15)
**IIa. Bone Remodeling + Oxidative Stress Markers**					
Ada Boost Classifier	0.65 (0.18)	0.59 (0.26)	0.65 (0.18)	0.63 (0.21)	0.62 (0.18)
**IIb. Subset of biomarkers: OPG, Catalase, SOD2, TRX1**					
Naive Bayes	0.68 (0.16)	0.70 (0.21)	0.68 (0.16)	0.70 (0.18)	0.66 (0.16)
**III. Integrated Feature Sets: IL-4, IFN-γ, SOD2, TRX1**					
Naive Bayes	0.71 (0.17)	0.66 (0.23)	0.71 (0.17)	0.72 (0.19)	**0.70 (0.18)**

## Data Availability

The data that support the findings of this study are openly available in Mendeley Data, V1, https://doi.org/10.17632/2wrkfmb99h.1.
